# Comparative analysis of the role of healthcare beliefs on childhood vaccination uptake among parents in Malaysia and Singapore during the COVID-19 pandemic

**DOI:** 10.3389/fpubh.2025.1470345

**Published:** 2025-04-01

**Authors:** Jia Ming Low, Erwin Jiayuan Khoo, Meow Keong Thong, Chloe Soo, Anh Phuong Tran, Le Ye Lee, Fook Choe Cheah

**Affiliations:** ^1^Department of Neonatology, Khoo Teck Puat – National University Children Medical Institute, National University Hospital, National University Health System, Singapore, Singapore; ^2^Department of Paediatrics, Yong Loo Lin School of Medicine, National University of Singapore, Singapore, Singapore; ^3^Department of Paediatrics, International Medical University, Kuala Lumpur, Malaysia; ^4^Center for Bioethics, Harvard Medical School, Harvard University, Boston, MA, United States; ^5^Department of Paediatrics, Faculty of Medicine, University of Malaya, Kuala Lumpur, Malaysia; ^6^M. Kandiah Faculty of Medicine and Health Sciences, Universiti Tunku Abdul Rahman, Kampar, Malaysia; ^7^Department of Paediatrics, Faculty of Medicine, Universiti Kebangsaan Malaysia (UKM), Kuala Lumpur, Malaysia; ^8^Neonatology Units, Hospital Canselor Tuanku Muhriz (HCTM) and Hospital Pakar Kanak-Kanak (HPKK), University Kebangsaan Malaysia (UKM), Kuala Lumpur, Malaysia

**Keywords:** COVID-19, health beliefs, paediatric vaccines, trust, vaccine hesitancy

## Abstract

**Introduction:**

The rollout of successful vaccination programs during the COVID-19 pandemic has been impeded worldwide by high rates of vaccine hesitancy. We investigated vaccine hesitancy rates in Malaysia and Singapore, and explored whether these rates were associated with parents’ health beliefs.

**Methods:**

A total of 226 Malaysian parents (MPs) and 635 Singaporean parents (SPs) participated in an online voluntary survey between November 2021 and August 2022.

**Results:**

MPs were younger and had more children compared to SPs. SPs were more likely to have received the COVID-19 vaccine than MPs, and less likely to delay vaccinations for their children. SPs displayed greater trust in information about vaccines, their children’s doctors and healthcare authorities than MPs. Despite the similarities in ethnography and geographic proximity, the prevalence of perceived parental vaccine hesitancy was higher in Malaysia than in Singapore; this was associated with differences in healthcare beliefs.

**Discussion:**

Beyond educational campaigns, strengthening community-based healthcare support, addressing misinformation, and fostering transparent communication from healthcare authorities may further enhance parental trust in vaccine.

## 1 Introduction

Vaccine hesitancy is a worldwide but complex phenomenon listed among the top ten threats to global health by the World Health Organization in 2019 (1). The COVID-19 pandemic has further compounded the various factors contributing to vaccine hesitancy, perhaps due to heightened public attention and scrutiny of news surrounding the accelerated development and approval of COVID-19 vaccines, as well as the potential spread of misinformation on social media. When the vaccines are offered to children, parents play an important role in making informed decisions for their child’s well-being.

Prior to the COVID-19 pandemic, both Malaysia and Singapore reported high overall national immunization rates of up to 95% (2, 3). Previous studies have reported that up to 8.0% of Malaysian parents had concerns about vaccine safety and side effects, driven by cultural and religious factors as well as misinformation (2, 4, 5). Similarly, in Singapore, localized pockets of hesitancy have been observed primarily for paediatric influenza and pneumococcal vaccines (6). This largely stemmed from the common misconception among both parents and healthcare workers that these vaccines were primarily required only when traveling overseas.

In Malaysia, COVID-19 childhood vaccination was first offered to teenagers aged 12 years and older from September 15, 2021, and subsequently to children younger than 12 years from February 3, 2022 (7). The brands of vaccines used in Malaysia were from Pfizer (61.2%), Sinovac (29.8%), AstraZeneca (7.9%), and Cansino (0.3%) (8). In Singapore, its Ministry of Health approved the use of the paediatric dose of the Pfizer-BioNTech/Comirnaty vaccine for teenagers in June 2021, and for children aged 5–11 years in December 2021. However, the uptake of the paediatric COVID-19 vaccination was significantly lower compared to the rate of adult and adolescent vaccination (9).

In facing the continuing waves of COVID-19, it is important to address the various factors contributing to vaccine hesitancy amongst parents in societies with diverse cultural and socioeconomic contexts. Malaysia and Singapore are two adjacent countries with similar diverse populations but differing economic wealth. Analyzing the results of vaccine hesitancy rates in these countries can provide valuable insights into common areas of interest, the different risk factors, and the development of solutions identified when one country performs better than the other in terms of vaccine acceptance. These findings can inform health care policies and communitarian decisions that shape appropriate public health interventions in the future.

The aim of this study was to determine and compare the healthcare beliefs among parents of eligible children for COVID-19 vaccination in Malaysia and Singapore during the COVID-19 pandemic. We also aimed to compare the social and demographic features of parents who perceived themselves as more vaccine hesitant, to determine parental trust in their healthcare systems, and to identify the preferred type of vaccine that they would have had considered for their children.

## 2 Methods

The online cross-sectional study was conducted from November 2021 to August 2022 through a secure electronic platform. A prospective, anonymous, and voluntary electronic survey modified from the previously developed “Measuring vaccine hesitancy: the development of a survey tool” was used (Supplementary Table 1), and it has been validated in a separate study (10). A translated version in Bahasa Melayu was utilized in Malaysia.

The study population consisted of parents of children who were hospitalized or attending outpatient clinics, as well as individuals who accessed the survey via links disseminated through official institutions’ social media platforms or official email channels. The study excluded parents less than 21 years of age who may not yet be eligible for the COVID-19 vaccines. Respondents did not receive any compensation for participating in the study.

The survey obtained demographic data of respondents and their children, as well as information on vaccine hesitancy, parental trust in the healthcare system, and preferred COVID-19 vaccine. Results were analyzed using chi-square analysis and multiple logistic regression analysis with Statistical Programme for Social Sciences (SPSS), IBM version 27. Statistical significance was defined when *p* < 0.05 in two-tailed tests.

The calculation of the sample size was based on the formula by Kish (11) for population survey using the StatCalc in EpiInfo software. With an estimated combined adult population in Malaysia and Singapore of 29,520,000 in 2022, an acceptable margin of error of 5%, an expected vaccine hesitancy prevalence of 50% (to optimize the sample size in view of the lack of previous studies for the current topic in the region) and, a design effect of 2.0 for possible clustering in the sampling, the calculated minimum sample size needed to achieve 95% confidence level was 768. The final sample size achieved was 861 – oversampling was done in anticipation of the high probability of non-response and missing data among the subjects.

The study was registered with the ethics review boards of the following institutions: Malaysia’s International Medical University (IMU) (reference no. 279/2021) and Universiti Kebangsaan Malaysia (UKM) (reference no. PPI/111/8/JEP-2021-824), as well as Singapore’s National Healthcare Group Domain Specific Review Board (NHG DSRB) (reference no. 2021/00900). Completion of the survey indicated participants’ consent to participate in the study.

## 3 Results

Supplementary Table 2 (unweighted) shows the demographic data of 861 parents who participated in the study, comprising 226 (26.2%) Malaysian parents (MPs) and 635 (73.8%) Singaporean parents (SPs). Due to the much higher non-response rate of the respondents in the Malaysian population than the Singapore population, post-stratification weight was determined based on the estimated adult population percentage ratio in both countries for the year 2022 (24,700,000 and 4,820,000, respectively). Based on the calculation explained by DeBell and Kronsnick (12). The post-stratification weight for respondents from Malaysia is 3.19 (0.8367/0.2625), whereas the post-stratification weight for respondents from Singapore is 0.22 (0.1633/0.7375). Due to this adjustment, all analyses would be shown with the unweighted and weighted results for comparison. For the weighted analyses in SPSS, the complex samples procedure was used whereby the sample weight was based on the post-stratification weight and the estimation of error was based on sampling with replacement design.

The findings show that MPs were generally younger, though the difference was not statistically significant (38.8 ± 8.8 vs. 39.1 ± 6.7 years, *p* = 0.64). Additionally, they had more children on average (2.4 ± 1.8 vs. 2.0 ± 0.8, *p* = 0.003) compared to SPs (Supplementary Table 2). There were also statistical differences noted in their educational level (*p* = 0.005) (Table 1), with more SPs having tertiary education than MPs (50.9% vs. 38.9%) (Supplementary Table 2). In terms of employment, more SPs were involved in the healthcare-related industries and information technologies, and were homemakers, while more MPs worked in the education sector (Supplementary Table 2; Table 1). With regards to their religious affiliations, SPs were more likely to identify themselves as Catholic, Christian, or with no religious belief, whereas MPs were more likely from the Muslim faith (Supplementary Table 2; Table 1). Comparing their marital statuses, a higher proportion of SPs were married (Supplementary Table 2; Table 1). This study found no significant difference in the proportions of parents who were able to work from home during the pandemic lockdown (Table 1).

**Table 1:** Multiple logistic regression analysis comparing socio-demographics of parents surveyed in Malaysia and Singapore.

	Unweighted^b^ (*n* = 848)		Weighted^c^ (estimated *n* = 841)	
Independent variables^a^	Adjusted prevalence odds ratio (being in the Singapore parents group)	*p* value of adjusted prevalence odds ratio (Wald’s test)	Adjusted prevalence odds ratio (being in the Singapore parents group)	*p* value of adjusted prevalence odds ratio (t-test)
Parent’s age in years	0.96	0.013	0.95	0.004
Education level		0.005		
Primary school or lower	Reference group	-	Reference group	-
Secondary school/Institute of Technical Education (ITE)	3.09	0.14	1.32	0.67
Junior college/polytechnic	2.06	0.32	1.24	0.71
University degree	1.92	0.37	1.35	0.60
Master’s degree or above	0.88	0.86	0.49	0.23
Area of work		0.007		-
Healthcare	Reference group	-	Reference group	0.34
Financial	0.94	0.8	0.67	0.72
Service industry	0.87	0.74	0.85	0.003
Manufacturing	5.63	0.011	6.71	0.10
Education	0.74	0.33	0.56	0.065
Energy and infrastructure	0.55	0.16	0.40	0.29
Information and communication technologies or biotechnology	2.09	0.087	1.62	0.013
Transport	2.57	0.19	4.30	0.88
Freelance/self-employed	1.37	0.51	0.90	0.18
Homemaker or unemployed	2.18	0.054	1.82	-
Religion		<0.001		
Buddhism	Reference group	-	Reference group	-
Catholic Christianity	14.44	0.013	20.3	0.001
Other Christianity	1.73	0.089	2.25	0.024
Hinduism	0.92	0.85	1.30	0.59
Muslim	0.12	<0.001	0.16	<0.001
Taoism	5.39	0.11	9.68	0.003
No religion	8.69	<0.001	14.13	<0.001
Others	0.38	0.29	0.89	0.89
Worked from home during lockdown period		0.011		
No	Reference group	-	Reference group	-
Partially	0.52	0.017	0.79	0.39
Yes	0.43	0.004	0.55	0.061
Marital status				
Married	Reference group	-	Reference group	-
Others	0.18	<0.001	0.17	<0.001
Model assumptions tested				
Ratio of cases to variables	Fulfilled		Fulfilled	
Adequacy of expected frequencies and power	Fulfilled		Fulfilled	
Absence of multicollinearity	Fulfilled		Fulfilled	
Model diagnostics				
Hosmer-Lemeshow goodness-of-fit (*p* value)	0.115 (Acceptable model fit)		Cannot be tested in SPSS	
Nagelkerke’s pseudo r^2^	0.42		0.40	
Area under the receiver operating characteristic curve (AUC ROC) (*p* value)	0.85 (<0.001) (moderately accurate model)		0.85 (<0.001) (moderately accurate model)	

Underlined *p* value: statistically significant result at *p* < 0.05.

a Final independent variables from the stepwise unweighted multiple logistic regression done based on the initial variables in Supplementary Table 2, whereby regrouping of categories in the variable was done if there was violation of the χ2 test assumption of minimum expected frequencies. If the issue of the violation persisted even if the regrouping was done or if it was not possible, then the variable was removed from the initial variables list.

b The unweighted multiple logistic regression was done by backward stepwise (Wald test criteria) method.

c The weighted multiple logistic regression was done by forced entry method based on the significant variables in the unweighted model.

SPs were more likely to have received the COVID-19 vaccine themselves (99.1% vs. 96.5%, *p* = 0.013) and perceived themselves as less vaccine hesitant (5.8% vs. 16.4%, *p* < 0.001 for overall hesitancy categories) (Supplementary Table 2; Table 2). They were also less likely to delay vaccination for their children, although non-significant statistically (19.4% vs. 26.5%, *p* = 0.507 for overall delay categories), and perceived vaccine-preventable diseases as more severe (62.7% vs. 56.2%, *p* = 0.008 for overall perception categories) (Supplementary Table 2). More than one-third of MPs were “very concerned” that the vaccines may not actually prevent the disease (36.3% vs. 27.2%, *p* = 0.010 for overall “concern” categories) (Supplementary Table 2; Table 2). Of interest, perceived vaccine hesitancy was lower among SPs than MPs, although a significantly higher percentage of SPs knew of someone with a bad reaction to the vaccine (46.3% vs. 35.4%, *p* = 0.005) (Supplementary Table 2; Table 2).

**Table 2:** Multiple logistic regression analysis comparing parental views on childhood immunizations in Malaysia and Singapore.

	Unweighted^b^ (*n* = 861)		Weighted^c^ (estimated *n*=861)	
Independent variables^a^	Adjusted prevalence odds ratio (being in the Singapore parents group)	*p* value of adjusted prevalence odds ratio (Wald’s test)	Adjusted prevalence odds ratio (being in the Singapore parents group)	*p* value of adjusted prevalence odds ratio (t-test)
Identified as vaccine hesitant for childhood vaccines (%)		0.001		
Not hesitant	Reference group	-	Reference group	-
Somewhat hesitant	1.12	0.59	0.99	0.98
Very hesitant	0.40	0.001	0.36	0.001
Level of concern that vaccine might not be able to prevent disease		0.062		
Not concerned	Reference group	-	Reference group	-
Somewhat concerned	0.70	0.11	0.75	0.23
Very concerned	0.55	0.018	0.58	0.046
Known someone with a bad reaction to a vaccine				
Yes	1.99	<0.001	1.87	0.001
No	Reference group	-	Reference group	-
The only reason why I consented for my child to receive vaccinations is because it is a mandated requirement for day-care/school		0.004		
Disagree	Reference group	-	Reference group	-
Ambivalent	0.54	0.004	0.50	0.004
Agree	0.54	0.005	0.51	0.004
I trust the information I received about vaccins.		<0.001		
Disagree	Reference group	-	Reference group	-
Somewhat agree/disagree	3.08	<0.001	3.38	<0.001
Agree	2.75	0.001	2.94	0.001
It is my role as a parent to question the need for vaccines administered to my child		<0.001		
Disagree	Reference group	-	Reference group	-
Ambivalent	3.77	<0.001	3.70	<0.001
Agree	3.75	<0.001	3.54	<0.001
I am able to openly discuss my concerns about vaccinations with my child’s doctor				
Yes	0.55	0.071	0.72	0.37
No	Reference group	-	Reference group	-
Model assumptions tested				
Ratio of cases to variables	Fulfilled		Fulfilled	
Adequacy of expected frequencies and power	Fulfilled		Fulfilled	
Absence of multicollinearity	Fulfilled		Fulfilled	
Model diagnostics				
Hosmer-Lemeshow goodness-of-fit *p* value	0.611 (acceptable model fit)		Cannot be tested in SPSS	
Nagelkerke’s pseudo r^2^	0.13		0.11	
Area under the receiver operating characteristic curve (AUC ROC) (*p* value)	0.70 (<0.001) (Moderately accurate model)		0.70 (<0.001) (moderately accurate model)	

Underlined *p* value: statistically significant result at *p* < 0.05.

a Final independent variables from the stepwise unweighted multiple logistic regression done based on the initial variables in Supplementary Table 3, whereby regrouping of categories in the variable was done if there was violation of the χ2 test assumption of minimum expected frequencies. If the issue of the violation persisted even if the regrouping was done or if it was not possible, then the variable was removed from the initial variables list.

b The unweighted multiple logistic regression was done by backward stepwise (Wald test criteria) method.

c The weighted multiple logistic regression was done by forced entry method based on the significant variables in the unweighted model.

The study also found that SPs were less likely to distrust the information that they received about vaccines (6.6% vs. 14.6%, *p* < 0.001 for overall “trust” categories) and were less likely to question the necessity of vaccinations for their child (5.5% vs. 12.8%, *p* = 0.001 for overall “agree” categories), compared to MPs (Supplementary Table 2; Table 2). However, most parents in both countries agreed that they were able to openly discuss their concerns about vaccinations with their child’s doctor. Approximately two-thirds of parents (65.5 and 65.2% in Malaysia and Singapore, respectively) agreed with the recommended childhood vaccination schedule by their government (Supplementary Table 2; Table 2).

As shown in Supplementary Table 3 (unweighted) and Table 3, 68.1% of MPs and 70.2% of SPs stated that they would give the COVID-19 vaccine to their child between 6–11 years of age (*p* = 0.556).

**Table 3:** Multiple logistic regression analysis of factors associated with vaccine hesitancy among parents in Malaysia and Singapore.

	Unweighted^b^ (*n* = 850)		Weighted^c^ (estimated *n* = 856)	
Independent variables^a^	Adjusted prevalence odds ratio (being in the very Hesitant of vaccination group)	*p* value of adjusted prevalence odds ratio (Wald’s test)	Adjusted prevalence odds ratio (being in the very Hesitant of vaccination group)	*p* value of adjusted prevalence odds ratio (t-test)
Family monthly income (SGD)		< 0.001		
Below $4,000	Reference group	-	Reference group	-
$4,000–$9,999	0.41	0.004	0.27	0.011
$10,000–$14,999	0.14	< 0.001	0.37	0.23
$15,000–$19,999	0.45	0.12	0.22	0.008
$20,000 or above	0.21	0.012	0.15	0.003
Marital status				
Married	Reference group	-	Reference group	-
Others (single, divorce, separated)	0.25	0.079	0.49	0.35
Do you trust your child’s doctor?				
Yes (somewhat trust, Fully trust)	0.12	0.002	0.09	0.006
No (distrust)	Reference group	-	Reference group	-
The healthcare system has managed the COVID-19 pandemic well				
Yes (agree, somewhat agree, fully agree)	0.41	0.018	0.54	0.22
No (do not agree, somewhat disagree)	Reference group	-	Reference group	-
Model assumptions tested				
Ratio of cases to variables	Fulfilled		Fulfilled	
Adequacy of expected frequencies and power	Fulfilled		Fulfilled	
Absence of multicollinearity	Fulfilled		Fulfilled	
Model diagnostics				
Hosmer-Lemeshow goodness-of-fit *p* value	0.770 (acceptable model fit)		Cannot be tested in SPSS	
Nagelkerke’s pseudo r^2^	0.16		0.18	
Area under the receiver operating characteristic curve (AUC ROC) (*p* value)	0.75 (<0.001) (moderately accurate model)		0.69 (<0.001) (less accurate model)	

Underlined *p* value: statistically significant result at *p* < 0.05.

a Final independent variables from the stepwise unweighted multiple logistic regression done based on the initial variables in Supplementary Table 4, whereby regrouping of categories in the variable was done if there was violation of the χ2 test assumption of minimum expected frequencies. If the issue of the violation persisted even if the regrouping was done or if it was not possible, then the variable was removed from the initial variables list.

b The unweighted multiple logistic regression was done by backward stepwise (Wald test criteria) method.

c The weighted multiple logistic regression was done by forced entry method based on the significant variables in the unweighted model.

A higher proportion of parents expressed a willingness to vaccinate their teenager, with 79.2% of MPs and 82.8% of SPs indicating that they would do so (*p* = 0.224) (Supplementary Table 3). A significantly greater proportion of SPs than MPs expressed trust in information, advice, and recommendations regarding COVID-19 vaccination from their children’s doctors or healthcare authorities (80.8% vs. 69.9%, *p* = 0.005 for overall “source” categories) (Supplementary Table 3 and Table 3). Furthermore, respondents from both countries rated mRNA vaccines as the most effective in terms of preventing deaths and hospital admission compared to other types of COVID-19 vaccines (45.6% for MPs vs. 60.6% for SPs, *p* < 0.001 for overall vaccine categories) (Supplementary Table 3; Table 3).

Table 4 (unweighted) shows a significantly higher proportion of perceived “very hesitant” parents in Malaysia compared to Singapore (*p* < 0.001). Furthermore, parents of Muslim faith exhibited a higher likelihood of perceived vaccine hesitancy compared to other religions (47.4% Muslims accounts for very hesitant group vs. 29.5% Muslims accounts for non-hesitant or somewhat hesitant group, *p* = 0.026 for overall religion categories). A notable association was also found between trust in the child’s doctor and vaccine hesitancy (*p* < 0.001), where parents who were fully trusting in their child’s doctor were less likely to be classified as “very hesitant.” Correspondingly, parents who disagreed with their government’s (*p* = 0.01) and healthcare system/department’s (*p* < 0.001) management of the pandemic were more likely to be “very hesitant” about vaccines (Table 4).

**Table 4:** Descriptive analyses of factors associated with perceived vaccine hesitancy among parents in Malaysia and Singapore.

	Unweighted			Weighted		
Variables	Not hesitant or somewhat hesitant parents, *n* = 787 unless otherwise stated (%)	Very hesitant parents, *n* = 74 unless otherwise stated (%)	*p*-value (χ^2^ test)	Not hesitant or somewhat hesitant parents, estimated *n* = 734 unless otherwise stated (%)	Very hesitant parents, estimated *n* = 126 unless otherwise stated (%)	*p*-value [weighted χ^2^ test (Rao-Scott adjusted)]
Country						
Malaysia	189 (24.0)	37 (50.0)	< 0.001	603 (82.1)*^##^*	118 (93.5)*^##^*	<0.001
Singapore	598 (76.0)	37 (50.0)		131 (17.9)*^##^*	8 (6.5)*^##^*	
Gender						
Female	506 (64.3)	46 (62.2)	0.72	390 (53.2)*^##^*	72 (57.5)*^##^*	0.61
Male	281 (35.7)	28 (37.8)		344 (46.8)*^##^*	54 (42.5)*^##^*	
Place of birth of parent						
Singapore	435 (55.3)	23 (31.1)	<0.001^$^	99 (13.4)*^##^*	5 (4.0)*^##^*	0.18
Malaysia	234 (29.7)	41 (55.4)		577 (78.6)*^##^*	113 (89.5)*^##^*	
India, Sri Lanka, Pakistan	40 (5.1)	5 (6.8)		27 (3.6)*^##^*	4 (3.2)*^##^*	
China, Taiwan, Hong Kong, Japan	21 (2.7)	3 (4.1)		5 (0.6)*^##^*	1 (0.5)*^##^*	
Other South-East Asia countries	36 (4.6)	2 (2.7)		11 (1.5)*^##^*	3 (2.7)*^##^*	
Australia, New Zealand, USA, European countries, South American countries	21 (2.7)	0 (0.0)		17 (2.2)*^##^*	0 (0.0)*^##^*	
Age of parent	*n*=783	*n*=72	0.65	*n*=722	*n*=120	0.61
≤30 years	64 (8.2)	7 (6.0)		82 (11.4)*^##^*	10 (8.7)*^##^*	
>30 years	719 (91.8)	65 (90.3)		639 (88.6)*^##^*	109 (91.3)*^##^*	
Education level						
Primary education or lower	13 (1.7)	2 (2.7)	0.068	18 (2.4)*^##^*	3 (2.7)*^##^*	0.49
Secondary school or ITE	63 (8.0)	12 (16.2)		61 (8.4)*^##^*	20 (16.2)*^##^*	
Junior college or polytechnic	126 (16.0)	11 (14.9)		135 (18.3)*^##^*	23 (18.4)*^##^*	
University degree	374 (47.5)	37 (50.0)		299 (40.7)*^##^*	53 (41.8)*^##^*	
Masters and above	211 (26.8)	12 (16.2)		222 (30.2)*^##^*	26 (20.9)*^##^*	
Religion						
Buddhism	145 (18.4)	13 (16.7)	0.026 ^$^	112 (15.3)*^##^*	12 (9.3)*^##^*	0.032
Catholic Christianity	41 (5.2)	3 (3.8)		12 (1.6)*^##^*	1 (0.5)*^##^*	
Other Christianity	165 (21.0)	8(10.3)		108 (14.6)*^##^*	5 (3.7)*^##^*	
Hinduism	48 (6.1)	5 (6.4)		40 (5.5)*^##^*	7 (5.6)*^##^*	
Islam	232 (29.5)	37 (47.4)		404 (55.1)*^##^*	100 (79.4*^##^*)	
Taoism	27 (3.4)	0 (0.0)		9 (1.2)*^##^*	0 (0.0)*^##^*	
No religion	123 (15.6)	8(10.3)		39 (5.3)*^##^*	2 (1.4)*^##^*	
Others	6 (0.1)	0 (0.0)		10 (1.4)*^##^*	0 (0.0)*^##^*	
Family monthly income (SGD)	*n* = 784	*n* = 74	< 0.001	*n* = 725	*n* = 126	0.003
Below $4,000	203 (25.9)	42 (56.7)		493 (68.0)*^##^*	113 (89.7)*^##^*	
$4,000–$9,999	269 (34.3)	20 (27.0)		133 (18.4)*^##^*	7 (5.8)*^##^*	
$10,000–$14,999	163 (20.8)	4 (5.4)		48 (6.6)*^##^*	4 (3.1)*^##^*	
$15,000–$19,999	67 (8.5)	5 (6.8)		27 (3.7)*^##^*	1 (0.9)*^##^*	
$20,000 or above	82 (10.5)	3 (4.1)		24 (3.3)*^##^*	1 (0.5)*^##^*	
Type of housing	*n* = 598	*n* = 37	0.024 ^$^	*n* = 132	*n* = 8	<0.001
Government HDB rental housing	25 (4.2)	3 (8.1)		6 (4.2)*^##^*	1 (8.1)*^##^*	
HDB 1–3 room flat	45 (7.5)	4 (10.8)		10 (7.5)*^##^*	1 (10.8)*^##^*	
HDB 4–5 room flat/executive condominium	346 (57.9)	20 (54.1)		76 (57.9)*^##^*	4 (54.1)*^##^*	
Maisonette	10 (1.7)	0 (0.0)		2 (1.7)*^##^*	0 (0.0)*^##^*	
Private condominium/landed property	171 (28.6)	8 (21.6)		38 (28.6)*^##^*	2 (21.6)*^##^*	
Others	1 (0.2)	2 (5.4)		1 (0.2)*^##^*	1 (5.4)*^##^*	
Marital status	*n* = 785	*n* = 74	0.67^$^	*n* = 728	*n* = 126	0.63
Single	16 (2.0)	2 (2.7)		36 (5.0)*^##^*	6 (5.1)*^##^*	
Married	746 (95.0)	72 (97.3)		663 (91.1)*^##^*	120 (94.9)*^##^*	
Divorced	17 (2.2)	0 (0.0)		16 (2.1)*^##^*	0 (0.0)*^##^*	
Separated	6 (0.8)	0 (0.0)		13 (1.8)*^##^*	0 (0.0)*^##^*	
Have you received your COVID-19 vaccination?						
Yes	780 (99.1)	67 (90.5)	< 0.001 ^$^	724 (98.6)*^##^*	110 (87.0)*^##^*	<0.001
No	7 (0.9)	7 (9.5)		10 (1.4)*^##^*	16 (13.0)*^##^*	
Trust in Child’s doctor	*n* = 785	*n* = 74	< 0.001			<0.001
Distrust	4 (0.5)	9 (12.2)		7 (0.9)*^##^*	23 (18.0)*^##^*	
Somewhat Trust	301 (38.3)	37 (50.0)		259 (35.6)*^##^*	62 (48.8)*^##^*	
Fully Trust	480 (61.1)	28 (37.8)		462 (63.5)*^##^*	42 (33.1)*^##^*	
My government had managed the pandemic well						
Do not agree	42 (5.3)	11 (14.9)	0.016 ^$^	72 (9.7)*^##^*	29 (23.1)*^##^*	0.18
Somewhat disagree	69 (8.8)	9 (12.1)		92 (12.6)*^##^*	14 (11.0)*^##^*	
Agree	41 (5.2)	5 (6.8)		45 (6.1)*^##^*	7 (5.6)*^##^*	
Somewhat agree	369 (46.9)	26 (35.1)		307 (41.8)*^##^*	44 (35.1)*^##^*	
Fully agree	266 (33.8)	23 (31.1)		219 (29.8)*^##^*	32 (25.2)*^##^*	
My country healthcare system/health department has managed the pandemic well						
Do not agree	19 (2.4)	13 (17.6)	< 0.001 ^$^	43 (5.8)*^##^*	36 (28.2)*^##^*	<0.001
Somewhat disagree	36 (4.5)	5 (6.8)		47 (6.3)*^##^*	4 (3.2)*^##^*	
Agree	66 (8.4)	7 (9.4)		80 (10.9)*^##^*	10 (8.3)*^##^*	
Somewhat agree	239 (30.4)	17 (23.0)		266 (36.3)*^##^*	33 (26.5)*^##^*	
Fully agree	427 (54.3)	32 (43.2)		299 (40.7)*^##^*	43 (33.8)*^##^*	

Underlined *p* value: statistically significant result at *p* < 0.05 (2-tailed); *Independent t–test; ** Weighted general linear model; ^Independent t–t-test with a Satterthwaite approximation for degree of freedom due to unequal variance; ^#^ Estimated mean ± Standard deviation; ^##^ Estimated frequency (%); ^$^Fisher’s exact test done due to violation of χ2 test assumption of minimum expected frequencies.

Based on the multiple logistic regression analysis (Table 5) (unweighted), vaccine hesitancy was associated with (i) the lower income group (i.e., compared to the $4,000 group, the other income groups have adjusted prevalence odds ratio below than 1); (ii) distrust of child’s doctor (i.e., those who trust their child doctor have adjusted prevalence odds ratio below than 1); and, (iii) perception that the healthcare system has not managed the COVID-19 pandemic well (i.e., those who agree that the healthcare system was successful have adjusted prevalence odds ratio below 1).

**Table 5:** Multiple logistic regression analysis of willingness to vaccinate their children against COVID-19, trust in the healthcare system and preferences for vaccine type.

	Unweighted^b^ (*n* = 861)		Weighted^c^ (estimated *n* = 861)	
Independent variables^a^	Adjusted prevalence odds ratio (being in the Singapore parents group)	*p* value of adjusted prevalence odds ratio (Wald’s test)	Adjusted prevalence odds ratio (being in the Singapore parents group)	*p* value of adjusted prevalence odds ratio (t-test)
Would you give the COVID-19 vaccine to your child between 6–11 years old?				
Yes	0.61	0.022	0.77	0.24
No	Reference group	-	Reference group	-
My government has managed the COVID-19 pandemic well				
Yes	1.88	0.032	2.28	0.012
No	Reference group	-	Reference group	-
The healthcare system has managed the COVID-19 pandemic well				
Yes	2.34	0.016	2.41	0.032
No	Reference group	-	Reference group	-
Which source of information about safety and effectiveness of COVID-19 vaccines do you trust most?		0.010		
Government & health department	Reference group	-	Reference group	-
Television and radio	0.42	0.002	0.34	0.001
Social Media (including Facebook) or YouTube and Blog	1.39	0.36	1.65	0.22
Messaging platforms (including WhatsApp, WeChat, Telegram platforms)	0.76	0.45	0.81	0.58
Which of the following vaccines do you think has the least side effects?		0.028		
Inactivated vaccines	Reference group	-	Reference group	-
Non-replicating viral vectors vaccines	0.39	0.030	0.31	0.007
mRNA vaccines	1.12	0.61	1.03	0.90
Protein subunit vaccines or DNA vaccine type	2.54	0.11	3.90	0.013
Which of the vaccines do you think is the most effective (i.e., best prevent you from dying or requiring hospital admission)?		<0.001		
Inactivated vaccines	Reference group	-	Reference group	-
Non-replicating viral vectors vaccines	0.33	0.019	0.39	0.045
mRNA vaccines	2.99	<0.001	3.45	<0.001
Protein subunit vaccines or DNA vaccine type	2.64	0.162	3.17	0.103
Model assumptions tested				
Ratio of cases to variables	Fulfilled		Fulfilled	
Adequacy of expected frequencies and power	Fulfilled		Fulfilled	
Absence of multicollinearity	Fulfilled		Fulfilled	
Model diagnostics				
Hosmer-Lemeshow goodness-of-fit (*p* value)	0.233 (acceptable model fit)		Cannot be tested in SPSS	
Nagelkerke’s pseudo r^2^	0.22		0.17	
Area under the receiver operating characteristic curve (AUC ROC) (*p* value)	0.74 (<0.001) (moderately accurate model)		0.74 (<0.001) (moderately accurate model)	

Inactivated vaccines (e.g., Sinovac, Sinopharm, Bharat), mRNA vaccines (e.g., Pfizer/BioNTech, Moderna, Curevac), Protein subunit (e.g., Novavaxx), DNA (e.g., ZyCoV-D); Underlined *p* value: statistically significant result at *p* < 0.05.

a Final independent variables from the stepwise unweighted multiple logistic regression done based on the initial variables in Supplementary Table 4, whereby regrouping of categories in the variable was done if there was violation of the χ2 test assumption of minimum expected frequencies. If the issue of the violation persisted even if the regrouping was done or if it was not possible, then the variable was removed from the initial variables list.

b The unweighted multiple logistic regression was done by backward stepwise (Wald test criteria) method.

c The weighted multiple logistic regression was done by forced entry method based on the significant variables in the unweighted model.

## 4 Discussion

This study findings suggest that there are significant differences in the COVID-19 vaccine health care beliefs among parents in the two neighboring Southeast Asian countries. Our results are aligned with other estimates from different countries in Asia where the level of parental vaccine hesitancy varies, reportedly ranging between 10.8 and 42.8% (10, 13–15).

Factors identified in our study showed that parental vaccination status, trust in their healthcare provider and system were key factors associated with differences in vaccine beliefs. These findings confirm the result of other authors. A cross-sectional nationwide survey of Malaysian parents also showed that parents’ history of COVID-19 vaccination was the strongest predictor of their willingness to vaccinate their children (7). Likewise, in Singapore, trust in the child’s doctors was rated more important than information obtained from social media despite the high media usage in the country (10).

As such, we recommend that in managing future pandemics, it is imperative for all primary health care providers to receive up to date accurate information for dissemination to the public during the clinic visits. Doing so will foster an environment of greater understanding and cooperation. It is also important that healthcare providers remain up to date and provide evidence-based reasoning with regards to vaccination strategies in the face of potential new variants.

Unsurprisingly, a critical factor undermining vaccine use was trust in healthcare systems (16). Parents might be aware of shortcomings in the healthcare systems, both currently and historically. There can also be suspicion that when financial gains are involved, it is not unreasonable for suspecting dishonesty from those producing, distributing or promoting the vaccine. This belief could potentially be influenced by profits generated from COVID-19 vaccination by the pharmaceutical companies (17). Recommendations for boosters, vaccinations in the paediatric population who have been relatively spared from the COVID-19 pandemic may have only exacerbated negative vaccine belief sentiments amongst some individuals. Furthermore, rapid changes to policy statements, masking and isolation requirements, make vaccine hesitant parents more sceptical of the need for vaccine and its reliability.

The results of this survey indicate that perceived parental vaccine hesitancy was more prevalent in Malaysia than Singapore. The surveyed MPs trust of their doctors and healthcare system did not rate as high as the SPs, with a higher proportion of the MPs themselves remained unvaccinated against COVID-19. This finding is consistent with previous research that suggests vaccine hesitancy is more common in developing countries where one of the reasons for this may be related to the level of health literacy (18). Dubov et al. identified four groups of vaccine-hesitant individuals, including the misinformed, uninformed, undecided and unconcerned (19). Identification would allow for tailored individualized interventions to each (19). For example, personal analogies may be more effective for the “misinformed” individual, while motivational interviewing may be better suited for the “undecided” cluster. The study also found that Muslim parents were more likely to be “very hesitant” towards COVID-19 vaccines than parents of other religions. This result may be attributed to the predominant Muslim faith among MPs although this finding is also consistent with another similar online cross-sectional study conducted in ten countries in Asia, Africa, and South America earlier in the pandemic (20). However, parents’ age, gender, educational level, and marital status were not significantly associated with vaccine hesitancy in this study. This contrasts with other research that reported these demographic factors to be associated with vaccine hesitancy (21, 22). A possible explanation for these results may be a preponderance of respondents from urban communities in this study.

Our findings reveal that parental vaccine hesitancy is driven by a complex interplay of parental concerns and beliefs. Apart from trust in health information and the healthcare system, the way parents perceive the severity of vaccine-preventable diseases can drive their decision making. Our results show that SPs, who are more likely to perceive these complications as severe, have lower levels of hesitancy. This may be attributed to a higher parental health literacy level in a generally more educated population. This perception may be reinforced by social norms and the influence of healthcare professionals, who strive to effectively convey the importance of vaccination.

It was also reflected in our survey that a large proportion of parents surveyed in both countries agreed with the recommended childhood vaccination schedule by their government whilst holding back on vaccinating their child against COVID-19. We speculate that some parents may view vaccinating against COVID-19 as separate and unrelated, possibly due to the novelty of the mRNA technology and the rapidity of the development. This concurs with other published literature which have also shown that vaccine beliefs are not a stable trait (23). Some may also wrongly classify the vaccine as “experimental” and may feel that they do not want to subject their child to an ongoing experiment even though monitoring of adverse events after FDA approval is a standard procedure for all pharmaceuticals (24). On the other spectrum of things and contrary to these perceptions, parents in this study rated mRNA vaccines as the “most effective” in terms of preventing deaths and hospital admission compared to other types of COVID-19 vaccines. The reason for this is not clear but it may have something to do with the efficacy data of mRNA vaccines from clinical trials and real-world studies. In contrast, 1 in 6 surveyed parent perceived inactivated and non-replicating viral vector vaccines are most effective in preventing death and hospital admission with almost 40% of the parents believed they have the least side effects. Such vaccines are used in some other developing countries for children, and perhaps by having more options available, this may increase the vaccine uptake.

To address this issue of vaccine-hesitancy, effective communication strategies that address key concerns from stakeholders are crucial. A systematic review of interventions has revealed that the most successful campaigns are those that are dialogue-based and multi-pronged (25). Healthcare policy makers should also leverage on routine childhood immunization visits at community health centres or private health facilities to engage and get the buy-in from this group of parents. Finally, infotainment products in various languages may be used as an outreach strategy to supplement these efforts and specific media campaigns can be optimized to the type of vaccine attitude amongst different individuals who might be unsure or willing (26).

Strengths of this study include a detailed comparative analysis exploring the role of healthcare beliefs on childhood vaccination uptake between two countries with similarities in ethnography and geographic proximity between these two countries. This study may provide insights in the event of future pandemics to help fill the gaps between public engagement and health care beliefs of parents when it comes to administering novel vaccines to their children in the light of a pandemic.

We acknowledge some of the limitations of this survey. Firstly, this study was static and may not capture the rapidly evolving reality in the science, understanding, disease statistics and vaccination coverage during this COVID-19 pandemic. Secondly, the rather small sample size of parents who participated, may not be entirely representative of the views of the population especially in the geographically vaster Malaysia. A larger study involving other Asian countries with diverse socioeconomic backgrounds may allow for more in depth examination of the factors that influence vaccine hesitancy, which is a global health problem. In addition, a deeper exploration of how public health initiatives shape parental attitudes towards vaccination would enhance this report. However, the aim of our study was to examine parental healthcare beliefs regarding COVID-19 vaccination. As such, our data did not include measures capturing the influence of different public health approaches. Lastly, compared to Malaysia, Singapore is characterized by a much smaller geographic area, higher per capita income, and centralized healthcare system. Thus, the vaccine supply chain may be more streamlined in Singapore, ensuring rapid and widespread access. Such robust infrastructure may have contributed to the higher levels of trust observed amongst Singaporean parents. In contrast, while Malaysia has made significant strides in its national immunization program, the country’s larger and more geographically diverse landscape may pose unique logistical challenges.

In conclusion, our study highlighted the importance of healthcare providers in promoting vaccine acceptance and addressing vaccine hesitancy, and the importance of effective communication and public health messaging in this respect. Despite the proximity of these two countries that share many ethnographic similarities, perceived parental vaccine hesitancy is more common in Malaysia than Singapore because of different healthcare beliefs. Increasing educational efforts and public health awareness campaigns may be an approach to increase parental trust to improve the uptake of COVID-19 vaccinations in the paediatric population. Future research should explore and identify effective strategies for promoting vaccine acceptance.

## Data Availability

The original contributions presented in the study are included in the article/Supplementary material, further inquiries can be directed to the corresponding author.

## References

[1] Matos C Gonçalves BA Couto MT. Vaccine hesitancy in the global south: Towards a critical perspective on global health. Glob Public Health. (2022) 17:1087–98. doi: 10.1080/17441692.2021.1912138 33843459

[2] Trading Economics (2025). Malaysia – Immunization, DPT (% of Children Ages 12–23 Months). Available online at: https://tradingeconomics.com/malaysia/immunization-dpt-percent-of-children-ages-12-23-months-wb-data.html (accessed February 26, 2025).

[3] Ministry of Health, Singapore (2025). Newsroom – National Childhood and Adolescent Immunisation [Parliamentary QA, 1 March 2016]. Available online at: https://www.moh.gov.sg/newsroom/national-childhood-and-adolescent-immunisation (accessed February 26, 2025).

[4] Wong LP Wong PF AbuBakar S. Vaccine hesitancy and the resurgence of vaccine preventable diseases: the way forward for Malaysia, a Southeast Asian country. Hum Vaccin Immunother. (2020) 16:1511–20. doi: 10.1080/21645515.2019.1706935 31977285 PMC7482793

[5] Kalok A Loh SYE Chew KT Abdul Aziz NH Shah SA Ahmad S. Vaccine hesitancy towards childhood immunisation amongst urban pregnant mothers in Malaysia. Vaccine. (2020) 38:2183–9. doi: 10.1016/j.vaccine.2020.01.043 32001070

[6] Zahari M Offeddu V Smith GJD Tam CC. Determinants of influenza and pneumococcal vaccine uptake among preschool children in Singapore. PLoS One. (2023) 18:e0285561. doi: 10.1371/journal.pone.0285561 37196045 PMC10191335

[7] Ng DLC Gan GG Chai CS Anuar NAB Sindeh W Chua WJ. The willingness of parents to vaccinate their children younger than 12 years against COVID-19: a cross-sectional study in Malaysia. BMC Public Health. (2022) 22:1265. doi: 10.1186/s12889-022-13682-z 35768789 PMC9241237

[8] COVIDNOW (2022). Vaccinations in Malaysia. Available online at: https://covidnow.moh.gov.my/vaccinations/ (accessed February 26, 2025).

[9] Ministry of Health, Singapore (2025). Newsroom – Expert Committee on COVID-19 vaccination recommends using the paediatric dose Pfizer-BioNTech/Comirnaty vaccine for children aged 5–11 years and extending booster vaccination to persons aged under 30 years [Press Releases, 10 December 2021]. Available online at: https://www.moh.gov.sg/news-highlights/details/expert-committee-on-covid-19-vaccination-recommends-using-the-paediatric-dose-pfizer-biontech-comirnaty-vaccine-for-children-aged-5-11-years-and-extending-booster-vaccination-to-persons-aged-under-30-years (accessed March 27, 2023).

[10] Low JM Soo CWT Phuong T Zhong Y Lee LY. Predicting vaccine hesitancy among parents towards COVID-19 vaccination for their children in Singapore. Front Pediatr. (2022) 10:994675. doi: 10.3389/fped.2022.994675 36299688 PMC9589407

[11] Kish L. Survey Sampling. Am Polit Sci Rev. (1965) 59:1025. doi: 10.1017/S0003055400132113

[12] DeBell M Krosnick JA. Computing weights for American national election study survey data. Ann Arbor, MI, and Palo Alto, CA: American National Election Studies (2009). ANES Technical Report series, no. nes012427).

[13] Ali M Ahmed S Bonna AS Sarkar AS Islam MA Urmi TA. Parental coronavirus disease vaccine hesitancy for children in Bangladesh: a cross-sectional study. F1000Res. (2022) 11:90. doi: 10.12688/f1000research.76181.2 35211297 PMC8837808

[14] Kuan CI. Vaccine hesitancy and emerging parental norms: A qualitative study in Taiwan. Sociol Health Illn. (2022) 44:692–709. doi: 10.1111/1467-9566.13446 35174516

[15] Huynh G Nguyen HTN Van Tran K An PL Tran TD. Determinants of COVID-19 vaccine hesitancy among parents in Ho Chi Minh City, Vietnam. Postgrad Med. (2022) 134:303–8. doi: 10.1080/00325481.2022.2044142 35188041

[16] Schmelz K Bowles S. Overcoming COVID-19 vaccination resistance when alternative policies affect the dynamics of conformism, social norms, and crowding out. Proc Natl Acad Sci USA. (2021) 118:e2104912118. doi: 10.1073/pnas.2104912118 34099578 PMC8237671

[17] Lee J (2022). Pfizer expects a record $100 billion in revenue this year, thanks to COVID-19 vaccines and treatments (Market Watch). Available online at: https://www.marketwatch.com/story/pfizer-expects-a-record-100-billion-in-revenue-this-year-thanks-to-covid-vaccines-and-treatments-11644356651 (accessed March 27, 2023).

[18] Jaafar N Perialathan K Krishnan M Juatan N Ahmad M Mien TYS. Malaysian Health Literacy: Scorecard Performance from a National Survey. Int J Environ Res Public Health. (2021) 18:5813. doi: 10.3390/ijerph18115813 34071455 PMC8197907

[19] Dubov A Distelberg BJ Abdul-Mutakabbir JC Beeson WL Loo LK Montgomery SB. Predictors of COVID-19 Vaccine Acceptance and Hesitancy among Healthcare Workers in Southern California: Not Just "Anti" vs. "Pro" Vaccine. Vaccines (Basel). (2021) 9:1428. doi: 10.3390/vaccines9121428 34960171 PMC8706436

[20] Harapan H Anwar S Yufika A Sharun K Gachabayov M Fahriani M. Vaccine hesitancy among communities in ten countries in Asia, Africa, and South America during the COVID-19 pandemic. Pathogens Glob Health. (2021) 116:236–43. doi: 10.1080/20477724.2021.2011580 PMC9132408 34928187

[21] Khatatbeh M Albalas S Khatatbeh H Momani W Melhem O Omari OA. Children's rates of COVID-19 vaccination as reported by parents, vaccine hesitancy, and determinants of COVID-19 vaccine uptake among children: a multi-country study from the Eastern Mediterranean Region. BMC Public Health. (2022) 22:1375. doi: 10.1186/s12889-022-13798-2 35850675 PMC9294741

[22] Limbu YB Gautam RK Pham L. The Health Belief Model Applied to COVID-19 Vaccine Hesitancy: A Systematic Review. Vaccines (Basel). (2022) 10:973. doi: 10.3390/vaccines10060973 35746581 PMC9227551

[23] Siegler AJ Luisi N Hall EW Bradley H Sanchez T Lopman BA. Trajectory of COVID-19 Vaccine Hesitancy Over Time and Association of Initial Vaccine Hesitancy With Subsequent Vaccination. JAMA Netw Open. (2021) 4:e2126882. doi: 10.1001/jamanetworkopen.2021.26882 34559232 PMC8463937

[24] National Archives and Records Administration (NARA) Office of the Federal Register (OFR) (2023). Code of Federal Regulations. Available online at: https://bookstore.gpo.gov/products/code-federal-regulations-2023 (accessed March 27, 2023).

[25] Jarrett C Wilson R O'Leary M Eckersberger E Larson HJ SAGE Working Group on Vaccine Hesitancy. Strategies for addressing vaccine hesitancy—A systematic review. Vaccine. (2015) 33:4180–90. doi: 10.1016/j.vaccine.2015.04.040 25896377

[26] Yoneoka D Eguchi A Nomura S Kawashima T Tanoue Y Murakami M. Identification of optimum combinations of media channels for approaching COVID-19 vaccine unsure and unwilling groups in Japan. Lancet Reg Health West Pac. (2022) 18:100330. doi: 10.1016/j.lanwpc.2021.100330 34927110 PMC8665235

