# Supplementary material for: Comparative analysis of the role of healthcare beliefs on childhood vaccination uptake among parents in Malaysia and Singapore during the COVID-19 pandemic
Source: Front Public Health. 2025 Apr 1;13:1470345. doi: 10.3389/fpubh.2025.1470345 (PMC11996874; doi:10.3389/fpubh.2025.1470345)
Supplement: Supplementary file 1 [file Table_1.DOCX]

Supplementary Material

# Supplementary Tables

Supplementary Table 1. Survey questions.

| **Category** | **Questions** |
| --- | --- |
| **Demographic Data** | - What is your gender? - What is your year of birth? - How many children do you have?   - How many of them are above 6 years old?   - How many of them are above 12 years old? - What is your education level? - What is your area of work? - What is your religion? - Where is your place of birth? - If you were not born in Singapore, how many years have you resided here? - Have you worked or studied overseas? If so, how many years were you working/ studying overseas? - What is your family monthly income in S$? - What type of housing do you live in? - What is your marital status? |
| **Change in routine due to COVID-19 pandemic** | - Did you have a change from the usual work schedule from the office to home during the period of lockdown? - Did you work from home during the periods of lockdown? |
| **COVID-19 vaccination status and vaccination behavior** | - Have you received your COVID-19 jab? If no, what are the reasons why you have not received a COVID-19 vaccine? - Are you fully vaccinated against COVID-19? If not, what is the reason? |
| **Digital device and media use** | - What is your daily average use of digital devices for this week? - What is the total amount of time you spent on social media this week? - How much time on average do you spend watching TV per day? - How often if ever do you get news and updates on COVID-19 outbreaks from each of the following places? (Facebook, YouTube, Twitter, Weibo, Instagram, others such as newspapers) - Please tell us how much of what you know about COVID-19, if anything, comes from (Facebook/ Twitter or WhatsApp messages, Newspapers or other print material like magazines/ health leaflets, Television)? |
| **Vaccine safety and efficacy beliefs** | - Do you agree with the following beliefs about vaccine safety and efficacy? - Children get more vaccines than are good for them. - Many of the illnesses vaccines prevent are severe. - It is better for children to develop immunity by getting sick than by getting a shot. - It is better for children to get fewer vaccines at the same time. - How concerned are you that your child might have a serious side effect from a vaccine? - How concerned are you that any one of the childhood vaccines may not be safe? - How concerned are you that a vaccine might not prevent the disease? - Do you know anyone who has had a bad reaction to a vaccine? |
| **Overall attitude and trust** | - Have you (or would you intend to) ever delayed having your child get a vaccine for reasons other than illness or allergy? - Do you agree that the recommended vaccine schedule is (or would be) a good idea for your child? - Do you agree to the following statements? - It’s my role as a parent to question vaccines. - If you had another infant today, would you want him/her to get the recommended shots? - Overall, how hesitant about childhood vaccines would you consider yourself to be? - Which of the following statements reflect your general attitude and trust towards vaccines? - The only reason I would have my child get vaccines is do that they can enter day-care or school. - I trust the information I receive about vaccines. - Are you able to openly discuss your concerns about vaccines with your child’s doctor? - All things considered, how much do you trust your child’s doctor? - Would you give the COVID-19 vaccine (mRNA) to your teenager? - Would you give the COVID-19 vaccine (mRNA) to your child between 6-11 years old (if any)? - If HAS/MOH licenses a vaccine for use in children ,12 years old, would you give the COVID-19 vaccine (mRNA) to your child between 6- 11 years? - Do you agree with the following statements? - My government has managed the current pandemic well. - My country health care system/ health department has managed the current pandemic well. - Which have been your main sources of information about the safety and effectiveness of the COVID-19 vaccines? (Government and Health Department information, Television and radio, YouTube and bloggers, social media and large groups, Friends and small groups) - Which of the following vaccine types do you think is safest? (Inactivated vaccines, non-replicating viral vectors, mRNA, Protein subunit, DNA) - Which of the following vaccine types do you think is most effective? (Inactivated vaccines, non-replicating viral vectors, mRNA, Protein subunit, DNA) - If you could choose any vaccine in the world which vaccine, would you select and why? |

Adapted from Larson HJ, Jarrett C, Schulz WS, et al. Measuring vaccine hesitancy: The development of a survey tool. *Vaccine*. 2015;33(34):4165-4175. doi:10.1016/j.vaccine.2015.04.037

**Supplementary Table 2.** Descriptive analyses of socio-demographics of parents surveyed.

|  |  | Unweighted |  |  | Weighted | | | |
| --- | --- | --- | --- | --- | --- | --- | --- | --- |
| Variables | **Malaysian Parents (MP)**  **(n=226) (%)** | **Singapore Parents (SPs)**  **(n=635) (%)** | **All Parents**  **(n=861) (%)** | ***p*-value**  **(χ^2^ test)** | **Malaysian Parents (MP)**  **(estimated n=721) (%)** | **Singapore Parents (SPs)**  **(estimated n=140) (%)** | **All Parents**  **(estimated n=861) (%)** | ***p*-value**  **(Weighted χ^2^ test [Rao-Scott Adjusted])** |
| Parent’s age in years (mean ± SD) | 38·8 ± 7·3 | 39·1 ± 6·7 | 39·0 ± 6·9 | 0·64* | 38·8 ± 12·9^#^ | 39·1 ± 3·1^#^ | 38·1 ± 12·0^#^ | 0·65** |
| Number of children (mean ± SD) | 2·4 ± 1·8 | 2·0 ± 0·8 | 2·1 ± 1·6 | 0·003^ | 2·4 ± 3·2^#^ | 2·0 ± 0·4^#^ | 2·3 ± 3·0^#^ | 0·003** |
| Number of children under 6 (mean ± SD) | 1·3 ± 1·4 | 1·1 ± 1·1 | 1·1 ± 1·2 | 0·041^ | 1·3 ± 2·5^#^ | 1·1 ± 0·5^#^ | 1·3 ± 2·3^#^ | 0·040** |
| Number of children under 12 (mean ± SD) | 0·6 ± 1·0 | 0·5 ± 0·9 | 0·5 ± 0·9 | 0·11^ | 0·6 ± 1·8^#^ | 0·5 ± 0·4^#^ | 0·6 ± 1·6^#^ | 0·10** |
| Education level   - Primary school or lower - Secondary school/ Institute of Technical Education (ITE) - Junior college/ Polytechnic - University Degree - Master’s Degree or above | 6 (2·7)  22 (9·7)  43 (19·0)  88 (38·9)  67 (29·6) | 9 (1·4)  53 (8·3)  94 (14·8)  323 (50·9)  156 (24·6) | 15 (1·7)  75 (8·7)  137 (15·9)  411 (47·7)  223 (25·9) | 0·034 | 19 (2·7) *^##^*  70 (9·7) *^##^*  137 (19·0) *^##^*  281 (38·9) *^##^*  214 (29·6) *^##^* | 2 (1·4) *^##^*  12 (8·3) *^##^*  21 (14·8) *^##^*  71 (50·9) *^##^*  34 (24·6) *^##^* | 21 (2·5) *^##^*  82 (9·5) *^##^*  158 (18·3) *^##^*  352 (40·9) *^##^*  248 (28·8) *^##^* | 0·021 |
| Area of work   - Healthcare - Financial - Service industry - Manufacturing - Education - Energy & Infrastructure - Biotechnology - Information and communication technologies - Transport - Freelance/Self-employed - Homemaker - Unemployed | 61 (27·0)  32 (14·2)  19 (8·4)  4 (1·8)  52 (23·0)  19 (8·4)  1 (0·4)  11 (4·9)  3 (1·3)  10 (4·4)  14 (6·2)  0 (0·0) | 202 (31·8)  65 (10·2)  39 (6·1)  40 (6·3)  83 (13·1)  22 (3·5)  9 (1·4)  56 (8·8)  19 (3·0)  33 (5·2)  66 (10·4)  1 (0·2) | 263 (30·5)  97 (11·3)  58 (6·7)  44 (5·1)  135 (15·7)  41 (4·8)  10 (1·2)  67 (7·8)  22 (2·6)  43 (5·0)  80 (9·3)  1 (0·1) | < 0·001^$^ | 195 (27·0) *^##^*  102 (14·2) *^##^*  61 (8·4) *^##^*  13 (1·8) *^##^*  166 (23·0) *^##^*  60 (8·4) *^##^*  3 (0·4) *^##^*  35 (4·9) *^##^*  10 (1·3) *^##^*  32 (4·4) *^##^*  45 (6·2) *^##^*  0 (0·0) *^##^* | 44 (31·8) *^##^*  14 (10·2) *^##^*  8 (6·1) *^##^*  9 (6·3) *^##^*  18 (13·1) *^##^*  5 (3·5) *^##^*  2 (1·4) *^##^*  12 (8·8) *^##^*  4 (3·0) *^##^*  7 (5·2) *^##^*  14 (10·4) *^##^*  1 (0·2) *^##^* | 239 (27·8) *^##^*  116 (13·5) *^##^*  69 (8·0) *^##^*  22 (2·5) *^##^*  184 (21·4) *^##^*  65 (7·6) *^##^*  5 (0·6) *^##^*  47 (5·5) *^##^*  14 (1·6) *^##^*  39 (4·6) *^##^*  59 (6·9) *^##^*  1 (<0·1) *^##^* | < 0·001 |
| Religion   - Buddhism - Catholic Christianity - Other Christianity - Hinduism - Muslim - Taoism - No religion - Others | 30 (13·3)  1 (0·4)  25 (11·1)  12 (5·3)  150 (66·4)  1 (0·4)  4 (1·8)  3 (1·3) | 128 (20·2)  43 (6·8)  148 (23·3)  41 (6·5)  119 (18·7)  26 (4·1)  127 (20·0)  3 (0·5) | 158 (18·3)  44 (5·1)  173 (20·1)  53 (6·1)  269 (31·2)  27 (3·1)  131 (15·2)  6 (0·7) | < 0·001 | 96 (13·3) *^##^*  3 (0·4) *^##^*  80 (11·1) *^##^*  38 (5·3) *^##^*  479 (66·4) *^##^*  3 (0·4) *^##^*  13 (1.8) *^##^*  9 (1·3) *^##^* | 28 (20·2) *^##^*  10 (6·8) *^##^*  32 (23·3) *^##^*  9 (6·5) *^##^*  26 (18·7) *^##^*  6 (4·1) *^##^*  28 (20·0) *^##^*  1 (0·5) *^##^* | 124 (14·4) *^##^*  13 (1·5) *^##^*  112 (13·0) *^##^*  47 (5·5) *^##^*  505 (58·6) *^##^*  9 (1·0) *^##^*  41 (4·7) *^##^*  10 (1·2) *^##^* | < 0·001 |
| Worked from home during lockdown period   - No - Partially - Yes | 59 (26·1)  73 (32·3)  94 (41·6) | 207 (32·6)  171 (26·9)  257 (40·5) | 266 (30·9)  224 (26·0)  351 (40·8) | 0·13 | 188 (26·1) *^##^*  233 (32·3) *^##^*  300 (41·6) *^##^* | 46 (32·6) *^##^*  38 (26·9) *^##^*  56 (40·5) *^##^* | 234 (27·2) *^##^*  271 (31·4) *^##^*  356 (41·4) *^##^* | 0·13 |
| Marital status   - Married - Others | 203 (89·8)  23 (10·2) | 615 (96·9)  20 (3·1) | 818 (95·0)  43 (5·0) | < 0·001 | 648 (89·8) *^##^*  73 (10·2) *^##^* | 135 (96·9) *^##^*  5 (3·1) *^##^* | 783 (91.0) *^##^*  78 (9.0) *^##^* | < 0.001 |

*Note: Underlined p value: statistically significant result at p<0·05 (2-tailed);*

**Independent t-test; ** Weighted general linear model;*

*^Independent t-t-test with a Satterthwaite approximation for degree of freedom due to unequal variance; ^#^ Estimated mean* ± *Standard deviation; ^##^ Estimated frequency (%);*

*^$^Fisher’s exact test done due to violation of χ2 test assumption of minimum expected frequencies.*

**Supplementary Table 3.** Descriptive analyses of parental views on childhood immunizations.

|  |  | Unweighted |  |  | Weighted | | | |
| --- | --- | --- | --- | --- | --- | --- | --- | --- |
| Variables | **Malaysian Parents (MP)**  **(n=226) (%)** | **Singapore Parents (SPs)**  **(n=635) (%)** | **All Parents**  **(n=861) (%)** | ***p*-value**  **(χ^2^ test)** | **Malaysian Parents (MP)**  **(estimated n=721) (%)** | **Singapore Parents (SPs)**  **(estimated n=140) (%)** | **All Parents**  **(estimated n=861) (%)** | ***p*-value**  **(Weighted χ^2^ test [Rao-Scott Adjusted])** |
| Received COVID-19 vaccination | 218 (96·5) | 629 (99·1) | 847 (98·4) | 0·013^$^ | 695 (96·5) *^##^* | 139 (99·1) *^##^* | 834 (96·9) *^##^* | 0·008 |
| Fully vaccinated against COVID-19 at time of survey | 217 (96·9) | 625 (99·2) | 842 (97·8) | 0·018^$^ | 692 (96·9) *^##^* | 138 (99·2) *^##^* | 830 (97·3) *^##^* | 0·011 |
| Identified as vaccine hesitant for childhood vaccines (%)   - Not hesitant - Somewhat hesitant - Very hesitant | 133 (58·8)  56 (24·8)  37 (16·4) | 424 (66·8)  174 (27·4)  37 (5·8) | 557 (64·7)  230 (26·7)  74 (8·6) | < 0·001 | 424 (58·8) *^##^*  179 (24·8) *^##^*  118 (16·4) *^##^* | 93 (66·8) *^##^*  38 (27·4) *^##^*  8 (5·8) *^##^* | 517 (60·1) *^##^*  217 (25·2) *^##^*  126 (14·7) *^##^* | < 0·001 |
| Perceived that it is better for children to acquire natural immunity from infections instead of receiving vaccinations   - Yes - No | 168 (74·3)  58 (25.7) | 423 (66·6)  212 (33·4) | 591 (68·6)  270 (31·4) | 0·032 | 536 (74·3) *^##^*  185 (25·7) *^##^* | 93 (66·6) *^##^*  47 (33·4) *^##^* | 629 (73·1) *^##^*  232 (26·9) *^##^* | 0·032 |
| Level of concern that vaccine might not be able to prevent disease   - Not concerned - Somewhat concerned - Very concerned | 41 (18·1)  103 (45·6)  82 (36·3) | 167 (26·3)  295 (46·5)  173 (27·2) | 208 (24·2)  398 (46·2)  255 (29·6) | 0·010 | 131 (18·1) *^##^*  329 (45·6) *^##^*  262 (36·3) *^##^* | 37 (26·3) *^##^*  65 (46·5) *^##^*  38 (27·2) *^##^* | 168 (19·5) *^##^*  394 (45·7) *^##^*  300 (34·8) *^##^* | 0·010 |
| Felt that children received more vaccines than necessary   - Disagree - Ambivalent - Agree | 84 (37·2)  77 (34·1)  65 (28·8) | 253 (39·8)  235 (37·0)  147 (23·1) | 337 (39·1)  312 (36·2)  212 (24·6) | 0·24 | 268 (37·2) *^##^*  246 (34·1) *^##^*  207 (28·8) *^##^* | 56 (39·8) *^##^*  52 (37·0) *^##^*  32 (23·1) *^##^* | 324 (37·6) *^##^*  298 (34·5) *^##^*  239 (27·9) *^##^* | 0·27 |
| Believed that it is better for children to receive fewer vaccinations at the same setting   - Yes - No | 174 (77·0)  52 (23·0) | 471 (74·2)  164 (25·8) | 645 (74·9)  216 (25·1) | 0·40 | 555 (77·0) *^##^*  166 (23·0) *^##^* | 104 (74·2) *^##^*  36 (25·8) *^##^* | 659 (76·5) *^##^*  202 (23·5) *^##^* | 0·40 |
| Known someone with a bad reaction to a vaccine   - Yes - No | 80 (35·4)  146 (64·6) | 294 (46·3)  341 (53·7) | 374 (43·4)  487 (56·6) | 0·005 | 255 (35·4) *^##^*  466 (64·6) *^##^* | 65 (46·3) *^##^*  75 (53·7) *^##^* | 320 (37·2) *^##^*  541 (62·8) *^##^* | 0·005 |
| The only reason why I consented for my child to receive vaccinations is because it is a mandated requirement for day-care / school.   - Disagree - Ambivalent - Agree | 82 (36·3)  72 (31·9)  72 (31·9) | 306 (48·2)  178 (28·0)  151 (23·8) | 388 (45·1)  250 (29·0)  223 (25·9) | 0·006 | 262 (36·3) *^##^*  230 (31·9) *^##^*  230 (31·9) *^##^* | 67 (48·2) *^##^*  39 (28·0) *^##^*  33 (23·8) *^##^* | 329 (38·2) *^##^*  269 (31·2) *^##^*  263 (30·5) *^##^* | 0·005 |
| Have you (or do you intend to) delayed vaccines for your child for any reasons apart from illness/allergies?   - Never - Occasionally - All the time | 137 (60·6)  63 (27·9)  26 (11·5) | 409 (64·4)  167 (26·3)  59 (9·3) | 546 (63·4)  230 (26·7)  85 (9·9) | 0·51 | 437 (60·6) *^##^*  201 (27·9) *^##^*  83 (11·5) *^##^* | 90 (64·4) *^##^*  37 (26·3) *^##^*  13 (9·3) *^##^* | 527 (61·2) *^##^*  238 (27·6) *^##^*  96 (11·1) *^##^* | 0·51 |
| Illnesses that childhood vaccines can prevent are severe (%)   - Disagree - Ambivalent - Agree | 24 (10·6)  75 (33·2)  127 (56·2) | 31 (4·9)  206 (32·4)  398 (62·7) | 55 (6·4)  281 (32·6)  525 (61·0) | 0·008 | 76 (10·6) *^##^*  239 (33·2) *^##^*  405 (56·2) *^##^* | 7 (4·9) *^##^*  45 (32·4) *^##^*  88 (62·7) *^##^* | 83 (9·7) *^##^*  285 (33·1) *^##^*  493 (57·2) *^##^* | 0·018 |
| Do you agree with the recommended childhood vaccination schedule by the government?   - Yes - Unsure - No | 148 (65·5)  53 (23·5)  25 (11·1) | 414 (65·2)  176 (27·7)  45 (7·1) | 562 (65·3)  229 (26·6)  70 (8·1) | 0·11 | 472 (65·5) *^##^*  169 (23·5) *^##^*  80 (11·1) *^##^* | 91 (65·2) *^##^*  39 (27·7) *^##^*  10 (7·1) *^##^* | 563 (65·4) *^##^*  208 (24·1) *^##^*  90 (10·4) *^##^* | 0·13 |
| I trust the information I received about vaccines.   - Disagree - Somewhat agree/disagree - Agree | 33 (14·6)  92 (40·7)  101 (44·7) | 42 (6·6)  318 (50·1)  275 (43·3) | 75 (8·7)  410 (47·6)  376 (43·7) | < 0·001 | 105 (14·6) *^##^*  293 (40·7) *^##^*  322 (44·7) *^##^* | 9 (6·6) *^##^*  70 (50·1) *^##^*  61 (43·3) *^##^* | 114 (13·3) *^##^*  363 (42·2) *^##^*  383 (44·5) *^##^* | 0·002 |
| It is my role as a parent to question the need for vaccines administered to my child.   - Disagree - Ambivalent - Agree | 29 (12·8)  58 (25·7)  139 (61·5) | 35 (5·5)  205 (32·3)  395 (62·2) | 64 (7·4)  263 (30·5)  534 (62·0) | 0·001 | 92 (12·8) *^##^*  185 (25·7) *^##^*  443 (61·5) *^##^* | 8 (5·5) *^##^*  45 (32·3) *^##^*  87 (62·2) *^##^* | 100 (11·6) *^##^*  230 (26·7) *^##^*  530 (61·6) *^##^* | 0·003 |
| I am able to openly discuss my concerns about vaccinations with my child’s doctor   - Yes - No | 211 (93·4)  15 (6·6) | 580 (91·3)  55 (8·7) | 791 (91·9)  70 (8·1) | 0·34 | 673 (93·4) *^##^*  48 (6·6) *^##^* | 128 (91·3) *^##^*  12 (8·7) *^##^* | 801 (93·0) *^##^*  60 (7·0) *^##^* | 0·34 |
| If you had another child, would you want him/her to receive all the recommended childhood immunizations?   - Yes - Ambivalent - No | 150 (66·4)  48 (21·2)  28 (12·4) | 419 (66·0)  159 (25·0)  57 (9·0) | 569 (66·1)  207 (24·0)  85 (9·9) | 0·23 | 479 (66·4) *^##^*  153 (21·2) *^##^*  89 (12·4) *^##^* | 92 (66·0) *^##^*  35 (25·0) *^##^*  13 (9·0) *^##^* | 571 (66·3) *^##^*  188 (21·9) *^##^*  102 (11·8) *^##^* | 0·24 |
| Do you trust your child’s doctor?   - Yes - No | 215 (96·0)  9 (4·0) | 631 (99·4)  4 (0·6) | 846 (98·5)  13 (1·5) | 0·001*^$^* | 686 (96·0) *^##^*  29 (4·0) *^##^* | 139 (99·4) *^##^*  1 (0·6) *^##^* | 825 (96·5) *^##^*  30 (3·5) *^##^* | <0·001 |

*Note: Underlined p value: statistically significant result at p<0*·*05 (2-tailed);*

**Independent t-test; ** Weighted general linear model;*

*^Independent t-t-test with a Satterthwaite approximation for degree of freedom due to unequal variance; ^#^ Estimated mean* ± *Standard deviation; ^##^* Estimated frequency (%);

*^$^Fisher’s exact test done due to violation of χ2 test assumption of minimum expected frequencies.*

**Supplementary Table 4.** Descriptive analyses of parents’ willingness to vaccinate their children against COVID-19, trust in the healthcare system and preferences for vaccine type.

|  |  | Unweighted |  |  | Weighted | | | |
| --- | --- | --- | --- | --- | --- | --- | --- | --- |
| Variables | **Malaysian Parents (MP)**  **(n=226) (%)** | **Singapore Parents (SPs)**  **(n=635) (%)** | **All Parents**  **(n=861) (%)** | ***p*-value**  **(χ^2^ test)** | **Malaysian Parents (MP)**  **(estimated n=721) (%)** | **Singapore Parents (SPs)**  **(estimated n=140) (%)** | **All Parents**  **(estimated n=861) (%)** | ***p*-value**  **(Weighted χ^2^ test [Rao-Scott Adjusted])** |
| Would you give the COVID-19 vaccine to your child between 6-11 years old?   - Yes - No | 154 (68·1)  72 (31·9) | 446 (70·2)  189 (29·8) | 600 (69·7)  261 (30·3) | 0·56 | 491 (68·1) *^##^*  230 (31·9) *^##^* | 98 (70·2) *^##^*  42 (29·8) *^##^* | 589 (68·5) *^##^*  271 (31·5) *^##^* | 0·56 |
| Would you give the COVID-19 vaccine to your teenager?   - Yes - No | 179 (79·2)  47 (20·8) | 526 (82·8)  109 (17·2) | 705 (81·9)  156 (18·1) | 0·22 | 571 (79·2) *^##^*  150 (20·8) *^##^* | 116 (82·8) *^##^*  24 (17·2) *^##^* | 687 (79·8) *^##^*  174 (20·2) *^##^* | 0·22 |
| My government has managed the COVID-19 pandemic well   - Yes - No | 166 (73·5)  60 (26·5) | 564 (88·8)  71 (11·2) | 730 (84·8)  131 (15·2) | < 0·001 | 530 (73·5) *^##^*  191 (26·5) *^##^* | 124 (88·8) *^##^*  16 (11·2) *^##^* | 654 (75·9) *^##^*  207 (24·1) *^##^* | < 0·001 |
| The healthcare system has managed the COVID-19 pandemic well   - Yes - No | 188 (83·2)  38 (16·8) | 600 (94·5)  35 (5·6) | 788 (91·5)  73 (8·5) | <0·001 | 600 (83·2) *^##^*  121 (16·8) *^##^* | 132 (94·5) *^##^*  8 (5·5) *^##^* | 732 (85·0) *^##^*  129 (15·0) *^##^* | < 0·001 |
| Which source of information about safety and effectiveness of COVID-19 vaccines do you trust most?   - Government & health department - Television & radio - YouTube and Blog - Social Media (including Facebook) - Messaging platforms (including WhatsApp, WeChat, Telegram platforms) | 158 (69·9)  28 (12·4)  3 (1·3)  15 (6·6)  22 (9·7) | 513 (80·8)  41 (6·5)  5 (0·8)  41 (6·5)  35 (5·5) | 671 (77·9)  69 (8·0)  8 (0·9)  56 (6·5)  57 (6·6) | 0·005^$^ | 504 (69·9) *^##^*  89 (12·4) *^##^*  10 (1·3) *^##^*  48 (6·6) *^##^*  70 (9·7) *^##^* | 113 (80·8) *^##^*  9 (6·5) *^##^*  1 (0·8) *^##^*  9 (6·5) *^##^*  8 (5·5) *^##^* | 617 (71·7) *^##^*  98 (11·4) *^##^*  11 (1·2) *^##^*  57 (6·6) *^##^*  78 (9·0) *^##^* | 0·007 |
| Which of the following vaccines do you think has the least side effects?   - Inactivated vaccines - Non-replicating viral vectors vaccines - mRNA vaccines - Protein subunit vaccines - DNA vaccine type | 86 (38·1)  31 (13·7)  103 (45·6)  2 (0·9)  4 (1·8) | 193 (30·4)  15 (2·4)  385 (60·6)  37 (5·8)  5 (0·8) | 279 (32·4)  46 (5·3)  488 (56·7)  39 (4·5)  9 (1·0) | <0·001^$^ | 274 (38·1) *^##^*  99 (13·7) *^##^*  329 (45·6) *^##^*  6 (0·9) *^##^*  13 (1·8) *^##^* | 43 (30·4) *^##^*  3 (2·4) *^##^*  85 (60·6) *^##^*  8 (5·8) *^##^*  1 (0·8) *^##^* | 317 (36·8) *^##^*  102 (11·9) *^##^*  414 (48·0) *^##^*  14 (1·7) *^##^*  14 (1·6) *^##^* | < 0·001 |
| Which of the vaccines do you think is the most effective (i.e., best prevent you from dying or requiring hospital admission)?   - Inactivated vaccines - Non-replicating viral vectors vaccines - mRNA vaccines - Protein subunit vaccines - DNA vaccine type | 48 (21·2)  38 (16·8)  135 (59·7)  2 (0·9)  3 (1·3) | 58 (9·1)  10 (1·6)  543 (85·5)  22 (3·5)  2 (0·3) | 106 (12·3)  48 (5·6)  678 (78·7)  24 (2·8)  5 (0·6) | <0·001^$^ | 153 (21·2) *^##^*  121 (16·8) *^##^*  431 (59·7) *^##^*  6 (0·9) *^##^*  10 (1·3) *^##^* | 13 (9·1) *^##^*  2 (1·6) *^##^*  119 (85·5) *^##^*  5 (3·5) *^##^*  1 (0·3) *^##^* | 166 (19·3)  123 (14·3)  550 (63·9)  11 (1·3)  11 (1·2) | < 0·001 |

*Note:*  Inactivated vaccines (e.g., Sinovac, Sinopharm, Bharat), mRNA vaccines (e.g., Pfizer/BioNTech, Moderna, Curevac), Protein subunit (e.g., Novavaxx), DNA (e.g., ZyCoV-D);

*Underlined p value: statistically significant result at p<0*·*05 (2-tailed);*

**Independent t-test; ** Weighted general linear model;*

*^Independent t-t-test with a Satterthwaite approximation for degree of freedom due to unequal variance; # Estimated mean* ± *Standard deviation; ##* Estimated frequency (%);

*$Fisher’s exact test done due to violation of χ2 test assumption of minimum expected frequencies.*
